# Genome‐wide characterization of *Phytophthora infestans* metabolism: a systems biology approach

**DOI:** 10.1111/mpp.12623

**Published:** 2018-01-30

**Authors:** Sander Y. A. Rodenburg, Michael F. Seidl, Dick de Ridder, Francine Govers

**Affiliations:** ^1^ Laboratory of Phytopathology Wageningen University, Wageningen 6708 PB the Netherlands; ^2^ Bioinformatics Group Wageningen University, Wageningen 6708 PB the Netherlands

**Keywords:** metabolic model, metabolism, oomycete, *Phytophthora infestans*, systems biology

## Abstract

Genome‐scale metabolic models (GEMs) provide a functional view of the complex network of biochemical reactions in the living cell. Initially mainly applied to reconstruct the metabolism of model organisms, the availability of increasingly sophisticated reconstruction methods and more extensive biochemical databases now make it possible to reconstruct GEMs for less well‐characterized organisms, and have the potential to unravel the metabolism in pathogen–host systems. Here, we present a GEM for the oomycete plant pathogen *Phytophthora infestans* as a first step towards an integrative model with its host. We predict the biochemical reactions in different cellular compartments and investigate the gene–protein–reaction associations in this model to obtain an impression of the biochemical capabilities of *P. infestans*. Furthermore, we generate life stage‐specific models to place the transcriptomic changes of the genes encoding metabolic enzymes into a functional context. In sporangia and zoospores, there is an overall down‐regulation, most strikingly reflected in the fatty acid biosynthesis pathway. To investigate the robustness of the GEM, we simulate gene deletions to predict which enzymes are essential for *in vitro* growth. This model is an essential first step towards an understanding of *P. infestans* and its interactions with plants as a system, which will help to formulate new hypotheses on infection mechanisms and disease prevention.

## Introduction

The growth and functioning of any living cell are governed by a complex interconnected set of biochemical reactions, comprehensively referred to as its metabolism (Nielsen, 2017). It is essential for cells to consume and break down nutrients taken from the environment, and to use the resulting basic building blocks to construct the molecules needed for life (nucleic acids, amino acids, lipids, etc.) and for survival (secondary metabolites). However, the many molecules in this system and the many parameters that govern the biochemical reactions make metabolism difficult to study. Systems biology was introduced as a method to study a biological system as a whole by capturing its behaviour in a mathematical abstraction, i.e. a model (Ideker *et al*., 2001). A model can provide insights into the response of a biological system to certain perturbations or stimuli (Bordbar *et al*., 2014). A widely studied class of models is that of genome‐scale metabolic models (GEMs), which simulate and predict the metabolic behaviour of a cell (Lewis *et al*., 2012), such as the nutrients it can assimilate and the molecules it can synthesize.

The foundation of a GEM is the set of biochemical reactions that may occur in a cell, often catalysed by enzymes. Hence, the identification of enzyme encoding genes in the genome of an organism can help to reconstruct an overview of its biochemical capabilities (O'Brien *et al*., 2015; Yilmaz and Walhout, 2016). In a metabolic model, every reaction is considered as a conversion of substrate metabolites into product metabolites that takes place at a specific rate. The stoichiometry represents the balance of metabolites within the reaction. In steady state, i.e. a situation in which the net metabolite concentrations do not change, the reaction rates are called fluxes. A class of methods called constraint‐based modelling can be used to simulate the distribution of these fluxes in certain conditions (Orth *et al*., 2010). A well‐known constraint‐based method is flux balance analysis (FBA), which calculates the optimal set of flux values for the entire GEM to attain a specific metabolic objective. Typically, this metabolic objective is the maximization of biomass production, a synonym for growth, but can also entail different objectives, for instance, the minimization of energy consumption or redox potential (García Sánchez and Torres Sáez, 2014).

To date, several semi‐automated GEM reconstruction methods and protocols have been proposed (Agren *et al*., 2013; Karp *et al*., 2009; Schellenberger *et al*., 2011; Thiele and Palsson, 2010; Thiele *et al*., 2014), and the development of central databases for metabolic pathways and models has made biochemical information widely available (Caspi *et al*., 2014; Kanehisa *et al*., 2015; King *et al*., 2016). Although, initially, GEM reconstruction was mainly limited to microbes (prokaryotes and simple eukaryotes), the available resources now allow for the reconstruction of GEMs for complex organisms such as mammals and higher plants (Dharmawardhana *et al*., 2013; Thiele *et al*., 2013; Yuan *et al*., 2016). Such models have also already been applied to understand the metabolic interactions between pathogens and hosts (Duan *et al*., 2013; Huthmacher *et al*., 2010; Peyraud *et al*., 2016). This can provide new hypotheses about a pathogen's infection strategy and may suggest novel control targets (Chavali *et al*., 2011; Sharma *et al*., 2017).

*Phytophthora infestans* is the causal agent of the devastating disease late blight on tomato and potato, posing an important threat to global food production. It belongs to the oomycetes, a class in the eukaryotic Stramenopile lineage that comprises many plant and animal pathogens. *Phytophthora infestans* is considered to be one of the model species for oomycetes (Haas *et al*., 2009). In the asexual life cycle of *P. infestans*, different stages can be distinguished (Judelson, 2017). When the mycelium starts to sporulate, it forms sporangia that are dispersed by wind and water. Sporangia either germinate directly, starting new infections, or develop into zoosporangia that release zoospores. The latter encyst on plant contact and germinate, thereby forming an appressorium at the tip, from which a penetration peg emerges that mediates entry into the epidermal cells of the host plant. Cell wall‐degrading enzymes are secreted that may facilitate the penetration process (Brouwer *et al*., 2014; Meijer *et al*., 2014). After penetration, hyphae colonize the mesophyll, where they grow intracellularly and form haustoria inside the host cells (Whisson *et al*., 2016). These feeding structures provide a large contact area with the host cytosol, enabling efficient exchange of molecules, to mediate further infection. Apart from the pathogen–host interactions at the protein level, it can be anticipated that an unknown combination of metabolites is taken up from the plant by the pathogen as nutrients.

*Phytophthora infestans* is able to assimilate a wide range of compounds (Hohl, 1991). For example, *in vitro*, *P. infestans* is able to grow on pea, rye or Henninger medium, which contains an undetermined mixture of various nutrients, such as amino acids, organic acids and lipids (Griffiths *et al*., 2003; Meijer *et al*., 2014). Many of the *Peronosporales*, the lineage that comprises the *Phytophthora* genus, are sterol and thiamine auxotrophs, which implies that these compounds must be acquired from the host (Dahlin *et al*., 2017; Gaulin *et al*., 2010; Judelson, 2012). Although sterols are highly beneficial for mycelial growth, they are not essential (Hohl, 1991). Conversely, thiamine is essential for growth. The nutrients that are taken up by the pathogen are converted into biomass and secondary metabolites. *Phytophthora infestans* forms various long‐chain polyunsaturated fatty acids, predominantly arachidonic and eicosapentaenoic acid (EPA) (Griffiths *et al*., 2003; Sun *et al*., 2013). The oomycete cell wall is composed of various sugar polymers, mainly 1,3‐ and 1,6‐β‐glucans and cellulose (Grenville‐Briggs *et al*., 2008). Notably, both the long‐chain polyunsaturated fatty acids and the cell wall glucans can elicit plant immune responses (Robinson and Bostock, 2015), but it is likely that, during infection, such responses are suppressed by secreted effector proteins.

Large transcriptional changes of genes encoding metabolic enzymes were observed during the asexual life cycle of *P. infestans* (Ah‐Fong *et al*., 2017), suggesting profound changes at the metabolic level. Notably, metabolic enzymes in general were down‐regulated in the sporangia and zoospores, and many metabolic processes (e.g. biosynthesis of various amino acids) were up‐regulated in cysts and during mycelial growth (Ah‐Fong *et al*., 2017; Grenville‐Briggs *et al*., 2005). Moreover, elevated expression *in planta* of various nutrient transporter genes suggests a rich influx of nutrients during infection (Abrahamian *et al*., 2016). Transcriptome studies have analysed the metabolism of *P. infestans* from a regulatory point of view. However, these studies do not consider post‐transcriptional regulation and metabolic reaction fluxes. A GEM can provide an overview of *P. infestans* metabolism and, at the same time, predict the functioning of primary metabolism as a system. Here, we propose a first GEM for *P. infestans*.

## Results and Discussion

### Draft model reconstruction

We identified all putative enzymes encoded in the *P. infestans* genome (Haas *et al*., 2009) by matching all predicted protein sequences to hidden Markov models (HMMs), trained on groups of orthologous proteins from the Kyoto Encyclopedia of Genes and Genomes (KEGG) Orthology (KO) database (Agren *et al*., 2013; Kanehisa *et al*., 2015). This is a particularly suitable method for the detection of distant orthologues, as conserved domains have a strong influence on the alignment score and thus this method is sensitive to conserved catalytic domains (Pearson, 2013). Roughly 32% (5856) of the 18140 predicted *P. infestans* proteins matched a KO group, but not every KO group represents a metabolic enzyme catalysing a biochemical reaction. In total, 1408 *P. infestans* genes were associated with 1569 different biochemical reactions, involving 1663 different metabolites. (Table S1, see Supporting Information)

*Phytophthora infestans* is able to assimilate a range of nitrogen compounds, preferably amino acids, but also inorganic forms, such as nitrate (Hohl, 1991). As a carbon source, *P. infestans* prefers glucose or sucrose, but can also utilize many mono‐ and disaccharides (Judelson, 2017). Early experiments determined that *P. infestans* can utilize a range of organic sulfur and phosphorus compounds, although more optimal growth rates were observed with inorganic sulfate and phosphate sources (Fothergill and Child, 1964). We added uptake reactions to the model for the minimal synthetic growth medium from the literature (Hohl, 1991), the simplest nutrient combination shown to yield *in vitro* growth: glucose, ammonia, phosphate, sulfate and thiamine. Next, we composed a pool of biomass precursor metabolites that must be produced to sustain life: all nucleotides, all 20 L‐type amino acids, energy carriers (ATP, GTP) and the cofactors Coenzyme‐A, NADH, NADPH and FADH_2_, which are generally essential for a eukaryotic cell (Nielsen, 2017). The exact relative abundance of biomass components has never been quantified for *P. infestans*; therefore, the aforementioned biomass metabolites were added to the model as substrates of a single artificial biomass reaction with equal stoichiometry. In addition, for the phospholipids and fatty acids detected in *P. infestans* (Griffiths *et al*., 2003), excretion reactions were included. The known cell wall components 1,3‐ and 1,6‐β‐glucan and cellulose are all polysaccharides for which glucose is the precursor metabolite.

We used FBA to calculate the flux through each reaction, optimizing for biomass production (Orth *et al*., 2010). To predict quantitative fluxes using FBA, it is required to provide an accurate biomass composition, maintenance ATP requirements, growth rates and species‐specific reaction constraints (Thiele and Palsson, 2010). Although the lack of detailed data on *P. infestans* metabolism currently impairs reliable quantitative flux predictions, we can nevertheless deploy FBA to interrogate the model for its connectivity and topology.

**Figure 1:**
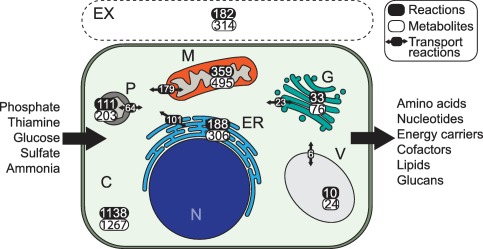
Schematic representation of a *Phytophthora infestans* cell with the number of reactions and metabolites per subcellular compartment and the number of transport reactions deduced from the model presented in this study. In this model, the nucleus (N) is not included as a separate subcellular compartment. C, cytosol; ER, endoplasmic reticulum; EX, extracellular space; G, Golgi complex; M, mitochondrion; P, peroxisome; V, vacuole.

Metabolic enzymes are located in various organelles, causing specific metabolic processes to take place in different parts of the cell. For example, the tricarboxylic acid (TCA) cycle typically occurs in the mitochondria (Zimorski *et al*., 2017). There is an extensive exchange of metabolites between subcellular compartments (Wanders *et al*., 2016). Obviously, the compartmentalization influences the connectivity of the reactions and the global behaviour of the model. Based on localization predictions by LocTree 3 (Goldberg *et al*., 2014), we expanded the model by dividing *P. infestans* proteins over seven subcellular compartments (Fig. 1). Reactions in the model were assigned to a particular compartment if at least one of the associated enzymes was predicted to localize there. LocTree has been trained on general eukaryotic sequences, which could influence the accuracy of our enzyme localization predictions. However, previous analyses using similar localization predictors have shown that proteins predicted to co‐localize are often also co‐expressed in *P. infestans* (Seidl *et al*., 2013). The cytosol contained 1138 reactions, whereas the mitochondria contained 359, which is approximately 15% of the total number of reactions in the model (Table 1). Of these 359, 160 (45%) were shared with the cytosol (Fig. S1, see Supporting Information). Notably, these shared reactions are part of various metabolic pathways, but a relatively large number (42) is linked to the fatty acid biosynthesis (FAB) pathway. The elongation of fatty acids can be governed by a single fatty acid synthase enzyme (EC 2.3.1.86). *P. infestans* has three gene copies for this enzyme, one of which is predicted to encode a mitochondrial isoform (PITG_18025). It has been reported that many eukaryotes have a highly conserved, independent mitochondrial FAB pathway that is crucial for development (Hiltunen *et al*., 2009; Kastaniotis *et al*., 2017). *Phytophthora* spp. are thought to store energy in fatty acid molecules to facilitate movement of zoospore flagellae (Judelson, 2017).

**Table 1:** Statistics of the *Phytophthora infestans* genome‐scale metabolic model (GEM) *iSR1301* and GEMs of other eukaryotic microbes.

	*Phytophthora infestans*	*Phaeodactylum tricornutum*	*Plasmodium falciparum*	*Leishmania donovani*	*Saccharomyces cerevisiae*
Reactions	2394	2156	1001	1135	1882
Transport	373	308	233	358	N/A
Cytosolic	1138	942	503	363	N/A
Mitochondrial	359	409	49	197	N/A
Metabolites	2685	1704	616	1135	1454
Genes	1301	1025	366	604	901
% of total	7.17	9.85	6.91	7.30	13.64
Model name	iSR1301	iLB1025	iTH366	iMS604	Yeast 7
Reference	This study	Levering *et al*. (2016)	Plata *et al*. (2010)	Sharma *et al*. (2017)	Aung *et al*. (2013)

In our model, the TCA cycle shares five reactions with the cytosol. One of these is catalysed by malate dehydrogenase (MDH, EC 1.1.1.37). *P. infestans* has two genes encoding MDH, one encoding an isoform of MDH shown to be active in mitochondria in *P. infestans* and the other encoding a cytoplasmic isoform (López‐Calcagno *et al*., 2009). Other mitochondrial reactions are part of various metabolic pathways, including FAB, fatty acid degradation (β‐oxidation) and even three glycolytic reactions, involving seven enzymes. The mitochondrial localization of these latter enzymes is probably a remnant of a secondary endosymbiosis event (Judelson, 2017).

### Model correction enables flux simulations

After initial reconstruction of the model, 153 invalid reactions (e.g. polymer reactions, see Methods) and 107 associated genes were removed from the model. The reconstructed metabolic model of *P. infestans* was initially unable to simulate growth (flux towards all biomass components) because of missing reactions or invalid reaction directionality constraints. This can be the result of an incomplete genome sequence or misannotations. We therefore performed a model gap‐filling optimization to find the minimal set of reactions in KEGG that must be added to the model to correct this (Table S2, see Supporting Information). This method proposed 16 additional reactions, and highlighted three reactions that must be reversed to allow for the production of all biomass precursors. Next, eight extra drain reactions were added to the model to satisfy the steady‐state constraint. Notably, no gap‐filling solutions were found for the production of the fatty acids EPA and behenic acid (a.k.a docosanoate), both of which are produced by *P. infestans* (Griffiths *et al*., 2003; Robinson and Bostock, 2015; Sun *et al*., 2013). This is caused by the lack of fatty acid reactions in KEGG, leaving multiple fatty acid reactions unconnected to other reactions (see KEGG map 01040).

To simulate the metabolite exchange between subcellular compartments, the model must also include intracellular transport reactions. Although nutrient transporters in *P. infestans* have been studied (Abrahamian *et al*., 2016; Grenville‐Briggs *et al*., 2010), hardly anything is known about the metabolites that are exchanged between the cytosol and subcellular compartments. The annotated substrates for transporter proteins are not specific, and are therefore hard to integrate into the metabolic model. Moreover, transporter substrates, such as those from the Transporter Classification Database (Saier *et al*., 2016), are not cross‐linked with other databases. To overcome these limitations, we performed an optimization to identify the most likely set of intracellular transport reactions to be added to the model to allow the production of all possible metabolites. We determined what metabolites could ultimately be produced by the model, after which we selected the minimal set of transport reactions between the cytosol and any compartment to allow for this (Fig. 1). The extracellular space (regarded as a subcellular compartment) was excluded from this optimization. The metabolism in this compartment is largely governed by cell wall‐degrading enzymes. As it is not possible to distinguish the origin of the metabolites, pathogen or host plant, we had to exclude the extracellular space in these analyses.

After all correction steps, 928 of the 2394 (39%) reactions in the model were able to carry flux based on the defined growth medium, 377 of which carried a non‐zero flux when we calculated the optimal fluxes for maximal biomass production (Table S1, see Supporting Information). Of the 2685 metabolites in the model, 809 could not be produced based on our defined growth medium, and may require additional nutrient uptake. By iteratively adding uptake reactions to the model for each of these metabolites, we can simulate whether the import of a specific metabolite would allow the production of additional metabolites (Table S2). This reveals unresolved gaps in the model that could have a technical cause, but may also hint at biological properties. For example, episterol is proposed as a compound that would enable the production of four other metabolites. This is striking as *Phytophthora* spp. lack sterol biosynthesis enzymes and depend on sterol acquisition from the host plant (Dahlin *et al*., 2017). Another proposed metabolite is tyramine, which would, upon import into the model, enable the production of six other metabolites. Tyramine is a product of the decarboxylation of tyrosine and, based on the genome annotation, *P. infestans* seems to lack the enzyme that catalyses this reaction, i.e. tyrosine decarboxylase (EC 4.1.1.25). However, a more precise examination of the genome sequence revealed an unannotated open reading frame (on supercontig 1.18, position 2365580–2367055) that probably encodes this enzyme.

### The metabolic model connects genomic and metabolic properties

We compared the properties of the *P. infestans* GEM (designated iSR1301; File S1, see Supporting Information) with GEMs of other eukaryotic microbes (Table 1). The size of our *P. infestans* model, in terms of integrated reactions and genes, is on the same order of magnitude as that of a recent GEM of *Phaeodactylum tricornutum*, a closely related diatom (Levering *et al*., 2016), although our model involves more metabolites. The sizes of the GEMs of the malaria parasite *Plasmodium falciparum* (Plata *et al*., 2010) and the leishmaniasis parasite *Leishmania donovani* (Sharma *et al*., 2017) are much smaller, but the proportion of genes in the model is similar to that of the *P. infestans* model (∼7% of the total number of genes). Although these numbers might be smaller because of the genome annotation quality and the level of model curation, they might also be a result of the loss of primary metabolic pathways, for which these parasites rely on nutrient import from their hosts (Dean *et al*., 2014; Gardner *et al*., 2002). Despite the fact that *P. infestans* has a similar parasitic lifestyle, a pattern of pathway loss is not reflected in the size of our model.

The relation of a gene to an enzyme and its associated reactions is called the gene–protein–reaction (GPR) association (Machado *et al*., 2016; Thiele and Palsson, 2010). A reaction can be associated with multiple enzymes (isozymes) and genes (paralogues). Conversely, one enzyme may present multiple catalytic domains, or may have a broad substrate specificity, which associates it with multiple reactions. This ‘many‐to‐many‐to‐many’ relationship holds information about the redundancy of enzyme encoding genes in a genome, but also about gene essentiality, and the metabolic robustness of an organism to perturbations and fluctuations in nutrient availability (Belda *et al*., 2012). In our model, 40.4% of the genes are associated with just a single reaction, and 44.9% of the reactions in the model are associated with a single gene, which makes the respective genes essential for specific metabolic tasks (Fig. S2, see Supporting Information). In comparison, for the *P. tricornutum* GEM, these numbers are higher (68.6% and 54.5%, respectively). The diatom model is presumably of higher quality, as most reactions are manually curated. However, it might also hint at less redundancy of metabolic enzymes.

### Stage‐specific models reflect reduced metabolic activity in sporangia and zoospores

It has been demonstrated that the integration of transcriptomics data into a metabolic model has the potential to unveil condition‐ or tissue‐specific metabolic activity (Agren *et al*., 2012; Becker and Palsson, 2008; Gatto *et al*., 2014; Huthmacher *et al*., 2010). We had access to the transcriptome data of four asexual life stages, i.e. mycelium, sporangia, zoospores and germinating cysts (C. Schoina *et al*., unpublished data), and deployed the iMAT algorithm (Shlomi *et al*., 2008) to predict stage‐specific metabolic models for these life stages. This algorithm considers binary gene expression, i.e. a gene can either be expressed or not. Subsequently, it finds the fluxes through the model, supported by the maximum number of expressed genes, independent of defined medium and biomass composition. This results in sub‐models for which all included reactions can carry flux. However, not all underlying genes have to be expressed. In other words, the resulting stage‐specific models are sets of reactions that correlate best with the expression of the underlying genes. These reactions are therefore most likely to be metabolically active. If a reaction is absent from a stage‐specific model, it is either absent because the expression of the associated genes is low, or because upstream reactions are absent. Comparing the sets of reactions in each stage‐specific model might reveal highly active life stage‐specific metabolic activity. The distribution of stage‐wise expression values for the genes in the model forms a slimmer distribution (with slightly higher mean) than that of the total set of genes, indicating that genes in the model are more uniformly expressed (Fig. S3a, see Supporting Information). To generate a sufficiently large contrast between the stage‐specific models, we set the binary gene expression threshold at 7.04 transcripts per million (TPM), the median of all expression values. Based on this threshold, genes were called expressed/not expressed, and life stage‐specific models were calculated. Fewer genes were considered to be expressed in the sporangium and zoospore stages than in mycelium and germinating cyst stages (Fig. S3b).

**Figure 2:**
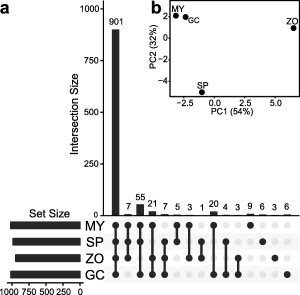
Stage‐specific models of *Phytophthora infestans* mycelium (MY), sporangia (SP), zoospores (ZO) and germinating cysts (GC). (a) Overlap of reaction content between the four stage‐specific models. The bars at the bottom left show the total numbers of reactions in each stage‐specific model. The connected bullets indicate the models that are compared, and the bars in the graph represent the number of reactions (intersection size, *y*‐axis) that overlap between the stage‐specific models. (b) Principal component analysis (PCA) of the stage‐wise presence/absence (1/0) of a reaction in the stage‐specific models.

The stage‐specific models for sporangium and zoospore stages contain fewer reactions in total (Fig. 2a), concordant with the observed general down‐regulation of many metabolic pathways in these stages (Ah‐Fong *et al*., 2017). The mycelium and germinating cyst models contain 1,021 and 1,017 reactions, respectively. Of these, 997 are shared, indicating that these models are highly similar. Although the majority of the reactions, i.e. a core set of 901 reactions, are shared between all four stage‐specific models, there are also obvious differences; 55 reactions are specifically absent from the zoospore model (hence present in the other three), 21 reactions are only absent from the sporangium model and 20 reactions are absent from the sporangium and zoospore models, but present in mycelium and germinating cyst models. A principal component analysis of stage‐wise reaction presence/absence (Fig. 2b) shows that the mycelium and germinating cyst models cluster relatively closely, whereas the sporangium and zoospore models are more isolated. In summary, our data reflect the regulatory changes that reroute the metabolism of *P. infestans* during each life stage, especially the transitions between mycelium/germinating cyst and sporangium/zoospore stages (Ah‐Fong *et al*., 2017).

To further interpret the presence/absence of reactions in the stage‐specific models, we looked at the associated metabolic pathways (Fig. S4, see Supporting Information). For instance, the mycelium model contains two unique reactions of the ‘Vitamin B6 metabolism’ pathway (KEGG R00173 and R00174), which represent the interconversion of pyridoxal (vitamin B6) to pyridoxal phosphate, an important cofactor for a large number of reactions, especially for the synthesis of amino acids (Percudani and Peracchi, 2003). The nitrogen metabolism pathway is represented by a core set of nine reactions, but the zoospore model lacks two reactions compared with mycelium and sporangia. Interestingly, these are reactions that contribute to glutamine and glutamate synthesis. Recently, the up‐regulation of nitrate transporters in zoospores has been reported (Ah‐Fong *et al*., 2017), which suggests an active nitrogen flux during this life stage. However, a reduced concentration of all amino acids was found in zoospores compared with other life stages (Grenville‐Briggs *et al*., 2005). As pointed out earlier, the expression of enzymes in the nitrogen metabolism pathway is highly dynamic and depends on the available nutrients (Abrahamian *et al*., 2016). Possibly, the nitrogen imported during the zoospore stage is stored and converted to amino acids at later life stages.

**Figure 3:**
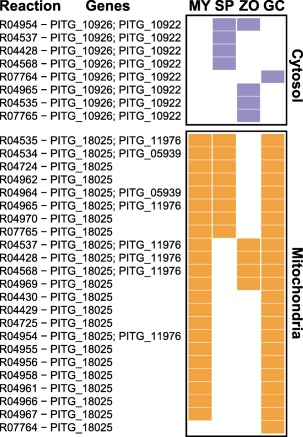
Fatty acid biosynthesis reactions in the stage‐specific models of *Phytophthora infestans* mycelium (MY), sporangia (SP), zoospores (ZO) and germinating cysts (GC). The presence/absence of a KEGG reaction (indicated by its ID followed by the associated gene IDs) is shown by filled/empty tiles, respectively, whereas mitochondrial and cytosolic reactions are shown in orange and purple, respectively.

We observed the largest contrast of stage‐wise reaction presence/absence in the FAB pathway (Fig. 3). A set of 10 reactions is present in the mycelium and germinating cyst models, and absent in the sporangium and zoospore models. Eight reactions are specifically absent in the zoospore model, but three other reactions are specifically present. The latter are all mediated by two cytosolic fatty acid synthases (PITG_10922 and PITG_10926), seemingly down‐regulated in other stages. Instead, the mitochondrial fatty acid synthase (PITG_18025) seems active in the mycelium and germinating cyst stages. It is likely that fatty acids are synthesized during hyphal stages, as zoospores are thought to use stored fatty acids as a nutrient source (Grant *et al*., 1988; Yousef *et al*., 2012). These data emphasize that fatty acids probably have an important role in *Phytophthora* zoospores. The three fatty acid synthase enzymes in *P. infestans* play a major role in the FAB process. Intriguingly, there could be a switch between cytosolic and mitochondrial FAB in zoospores. An unanticipated finding, reported by Ah‐Fong *et al*. (2017) and based on our model, is that fatty acid degradation (β‐oxidation) is not pronounced in the zoospore stage, despite the predicted role of fatty acids in zoospore motility (Judelson, 2017).

### Gene deletion simulations propose metabolic vulnerabilities

**Figure 4:**
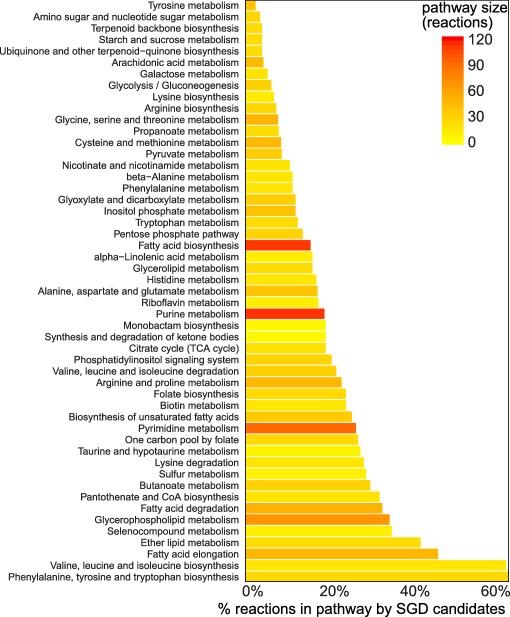
Gene deletion simulations in the *Phytophthora infestans* model. Percentage of reactions in each KEGG pathway that can be knocked out by a single gene deletion (SGD), disabling the production of at least one biomass precursor. The colours of the bars scale with the absolute numbers of reactions found in our model in a particular KEGG pathway (inset top right).

We investigated what effect gene deletions could have on the primary metabolism of *P. infestans*. By removing single genes from the model, one or more of the associated reactions in the model may be disabled. If such reactions are essential for the production of any of the biomass precursors, these deletions disable growth, i.e. the mathematical solution of the model becomes infeasible (O'Brien *et al*., 2015), making such genes interesting candidates for further study. We performed single gene deletion (SGD) simulations of all genes in the model, which suggested that 72 genes would disable growth by disabling the production of one of the essential biomass precursors (Table S2, see Supporting Information). These genes were associated with 285 reactions in various metabolic pathways (Fig. 4). The pathways ‘phenylalanine, tyrosine and tryptophan biosynthesis’ (17 of 26 reactions vulnerable to SGD) and ‘valine, leucine and isoleucine’ (11/17) were by far the most vulnerable pathways. Notably, the fatty acid degradation pathway was also delicate (17/111). In contrast, the most robust pathways were ‘tyrosine metabolism’ (1/39) and ‘amino sugar and nucleotide sugar metabolism’ (1/27).

There are numerous examples of the application of this method to suggest drug targets in pathogens (Hartman *et al*., 2014; Kaltdorf *et al*., 2016; Plata *et al*., 2010; Sharma *et al*., 2017; Yizhak *et al*., 2013), thus suggesting that the identified enzymes in *P. infestans* represent interesting candidates for further study. To confirm that these enzymes are essential for viability, ideally the encoding genes must be deleted or targeted by site‐directed mutagenesis. However, *P. infestans* is a diploid or polyploid organism, and making gene knock‐outs is not (yet) a straightforward procedure. However, gene silencing is feasible and has been applied to study the function of several genes involved in pathogenesis or signal transduction. The first functional study dealing with genes involved in primary metabolism was published only recently. Abrahamian *et al*. (2016) silenced the genes encoding nitrate and nitrite reductase, and their results suggested a role of these genes in virulence. Although both genes are included in our model, they were not marked as essential based on the SGD simulations, suggesting alternative routes for nitrogen metabolism. Our simulations are of course based on *in vitro* growth conditions, whereas, in its natural habitat, *P. infestans* mainly resides *in planta*. It is thought that *P. infestans* imports larger, organic nutrients, such as amino acids, during infection (Abrahamian *et al*., 2016).

## Concluding Remarks

Here, we present, to our knowledge, the first GEM for the oomycete *P. infestans*, reconstructed mostly *in silico*, based on reactions found in KEGG. The aim of this study was not to provide a fully quantitative model, but rather to provide a broad overview of cellular metabolism, related to its genome. We optimized the model to be able to convert a minimal pool of nutrients into a set of minimal biomass precursors established from the literature. Our model contributes to an understanding of the metabolism of *P. infestans*. However, even after gap‐filling, the fatty acids EPA and behenic acid could not be generated from the model, because of missing reactions in KEGG. This underscores the limitations of using KEGG solely as a resource, which may be overcome by manual refinement of the FAB pathways from different databases, such as MetaCyc or BRENDA (Caspi *et al*., 2014; Placzek *et al*., 2017). In fact, the BRENDA database holds information on an omega‐3 desaturase able to convert arachidonic acid into EPA (EC 1.14.19.25). This exact enzyme of *P. infestans* has recently been proven to be capable of catalysing this reaction in yeast (Yilmaz *et al*., 2017). We also revealed one unannotated tyrosine decarboxylase gene in the *P. infestans* genome, emphasizing the fact that the genome annotation of the reference genome published by Haas *et al*. (2009) needs to be revisited. On the other hand, it demonstrates that this model can be used to aid the discovery of unannotated genes. Obviously, the model will improve when a more accurate genome annotation becomes available, and future versions of the model should help us to address the shortcomings encountered in this study. In addition, an experimentally assessed biomass composition, transporter integration, inclusion of species‐specific reaction constraints and growth rates may be used to improve the accuracy of this model. The absence of these data restrains us from making quantitative predictions, such as growth rates or the influences of different nutrients.

Life stage‐specific models provide a direct functional context for transcriptome data by predicting the behaviour of *P. infestans* metabolism under the influence of stage‐wise gene expression, and are an alternative for the enrichment methods typically employed in metabolic pathway analyses. Approaching transcriptomic data from a functional point of view may emphasize certain features that are otherwise easily overlooked (e.g. FAB). By building this model, we can identify genes that have an essential role when converting simple nutrients to the building blocks of life. The metabolic model reconstructed here provides a scaffold for future genome‐wide systems biology approaches to characterize the metabolism of *P. infestans*, and is an essential first step towards an integrative model of *P. infestans*–host interactions.

## Methods

### Draft reconstruction

We re‐implemented the *getKEGGModelForOrganism* method from the RAVEN Toolbox (Agren *et al*., 2013) to improve performance and to incorporate minor adaptations. Briefly, HMMs were trained on orthologous enzyme sequences derived from the KO database, Release 2015‐11‐23. We constructed multiple sequence alignments (MSAs) of all eukaryotic protein sequences in every KEGG orthologous group using MAFFT version 7.273 (Katoh and Standley, 2013), using the ‘localpair’ mode for local alignments. For performance reasons, the number of sequences used in an MSA was capped at 100, in which case we selected a random subset of sequences in the KO group. If fewer than 20 eukaryotic sequences were included in a KO group, we constructed MSAs for prokaryotic sequences in the respective KO group, maintaining the same rules. We used *hmmbuild* from the HMMER package version 3.1b (Eddy, 1998) to train HMMs on the MSAs. By using *hmmsearch*, we matched the HMMs to the protein sequences of *P. infestans* strain T30‐4 (Haas *et al*., 2009), downloaded on 25 July 2015 from the BROAD Institute website (https://www.broadinstitute.org), currently hosted at the National Center for Biotechnology Information (NCBI, bioproject 17665). Default parameters were maintained for hmmbuild and hmmsearch, and an E‐value threshold of 10^−20^ was applied for hmmsearch. Similar to the *getKEGGModelForOrganism* function of the RAVEN Toolbox (Agren *et al*., 2013), we performed two pruning steps, but we applied slightly stricter thresholds. First, any protein match to a KO group was removed if $\frac{\log\left(E\right)}{\log{(E}_{bestKO})}<0.9$, where *E* represents the E‐value of the respective protein to a KO group, and *E*_bestKO_ represents the E‐value of the best‐matching KO group for that protein. In other words, protein hits are often removed if they have a better match to another KO group. Second, any protein match to a KO group was removed if $\frac{\log\left(E\right)}{\log\left(E_{bestProt}\right)}<0.5$, where *E* again represents the E‐value of the respective protein match to a KO group, and *E*_bestProt_ represents the lowest E‐value of any protein to this KO group. This reduces the number of matches per KO group to reduce the number of false positives, as there is clearly a better matching protein. Subsequently, we retrieved all KO annotations of *P. infestans* from KEGG (organism ID ‘pif’). The combined set of matched KO groups was used to retrieve all associated reactions and metabolites from KEGG. Consequently, each reaction in the model was associated with a number of *P. infestans* genes. We hypothesized that each of the genes associated with a reaction is able to catalyse it. We did not consider enzyme complexes, which together would fulfil a single enzymatic task. Reactions were removed automatically if their stoichiometry was undefined (e.g. ‘1,3‐beta‐D‐glucan(*n*) + UDP‐D‐glucose <=> 1,3‐beta‐D‐glucan(*n* + 1) + UDP’), and if the same metabolite ID occurred at both sides of the reaction arrow, which implies a polymer reaction (e.g. ‘UTP + RNA <=> diphosphate + RNA’). In addition, reactions that were associated with metabolites containing the substrings ‘acceptor’, ‘donor’, ‘tRNA’, ‘enzyme’, ‘aglycon’ and ‘fatty acid’ were removed.

### Model correction

We predicted the subcellular location of *P. infestans* proteins using LocTree 3 (Goldberg *et al*., 2014), and subsequently distributed the associated reactions of the model over the cellular compartments. We selected seven compartments for our model: cytosol, extracellular space, mitochondria, endoplasmic reticulum, Golgi complex, peroxisome and vacuole. Reactions associated with enzymes with transmembrane predictions (plasma or intracellular membranes) were assigned to respective compartments on both sides of the membrane, as it is unclear where the catalytic domain is localized. Proteins assigned to any other than our seven compartments were assigned to the cytosol. Next, all proteins that were predicted to be secreted by Raffaele *et al*. (2010) were assigned to the extracellular space compartment.

The initially reconstructed model was exported to SBML and Microsoft Excel format using CobraPy v0.4.1 (Ebrahim *et al*., 2013). For the next steps, we imported the model into MATLAB (R2015b) using the RAVEN Toolbox v1.8 (Agren *et al*., 2013). We used Gurobi v7.0.1 (http://www.gurobi.com/) to solve the (mixed‐integer) linear programs.

Nutrient uptake and biomass reactions were added to the model by applying the *fillGaps* function from the RAVEN Toolbox to propose gap solutions from KEGG, by constraining biomass production to a positive flux. This method implements the SMILEY algorithm (Rolfsson *et al*., 2011), including all reactions from a universal set of reactions (in this case KEGG), and subsequently minimizing the flux through these. Prior to this, we temporarily removed the extracellular space compartment to prevent gap solutions here, and added all possible transport and excretion reactions to the model. During gapfilling, we allowed the net production of metabolites, whereafter we added drain reactions (allowing excretion) for unbalanced metabolites to enable steady‐state solutions of the model at this point. To predict the set of transport reactions between compartments, we assessed which metabolites can ultimately be produced by the model. Then, we constrained a positive flux for the production of these metabolites, and we removed the transport reactions that did not carry flux.

After these correction steps, we used the function *solveLP* from the RAVEN Toolbox to solve the linear programs of FBA, and to obtain the flux distribution for maximal biomass production. We applied the function *checkProduction* to detect metabolite gaps in the model. This method checks which metabolites are not producible (blocked) from the model, and then iteratively adds uptake reactions for these metabolites, to see whether the uptake of these metabolites unblocks metabolites elsewhere in the model.

### Stage‐specific models

We used RNA sequencing data (C. Schoina *et al*., unpublished data) to quantify gene expression in four *in vitro* life stages of *P. infestans*, i.e. mycelium, sporangia, zoospores and germinating cysts. Gene expression of *P. infestans* strain T30‐4 (Haas *et al*., 2009) was quantified using Kallisto v0.42.4 (Bray *et al*., 2016), which expresses mRNA abundance in TPM. This unit represents the number of reads aligned to transcript sequences, normalized for transcript length and sequencing depth, scaled by a million. We determined a binary expression threshold to define whether or not a gene is expressed, for which we used the median of all expression values over the four life stages. We decomposed the biomass and other excreted compounds (lipids, etc.) into separate excretion reactions, and used the iMAT algorithm (implemented in the function *createTissueSpecificModel*) incorporated in the COBRA Toolbox (Schellenberger *et al*., 2011; Shlomi *et al*., 2008) to derive stage‐specific models.

### Gene knockout simulations

We used the function *findGeneDeletions* from the RAVEN Toolbox to predict the genes that would, upon deletion, disable biomass flux. This method first selects reactions that are supported by a single gene, and then iteratively constrains the flux of these reactions to zero. A gene is marked as essential if the linear program of FBA becomes infeasible after deletion, i.e. when no flux through the biomass reaction is possible.

## Supporting information

Additional Supporting Information may be found in the online version of this article at the publisher's website:

**Fig. S1** Reactions in the *Phytophthora infestans* model per subcellular compartment, and overlap in reaction content between different subcellular compartments. These include the cytosol (cyto), mitochondrion (mito), extracellular space (extr), endoplasmic reticulum (ER), peroxisome (pero), Golgi complex (golg) and vacuole (vacu). The bars at the bottom left show the total numbers of reactions in each subcellular compartment. The connected bullets indicate the compartments that are compared, and the bars in the graph represent the number of reactions (intersection size, *y*‐axis) that overlap between the compartments.

**Fig. S2** Frequencies of gene numbers per reaction (a) and reaction numbers per gene (b) in the *Phytophthora infestans* model.

**Fig. S3** Transcriptome data of *Phytophthora infestans* in relation to stage‐specific metabolic models. (a) Distributions of transcripts per million (TPM) expression values of all *P. infestans* genes (background) and the genes in the model, combined from four life stages. The red line indicates the TPM threshold set to distinguish expressed/non‐expressed genes in the model. (b) The percentages of all *P. infestans* genes (background) and the genes in the model for which gene expression in each life stage exceeds the TPM threshold. MY, mycelium; SP, sporangia; ZO, zoospores; GC, germinating cysts.

**Fig. S4** Numbers of reactions per KEGG pathway that are shared between the *Phytophthora infestans* life stage‐specific models of mycelium (MY), sporangia (SP), zoospores (ZO) and germinating cysts (GC). The colours of the tiles scale to the relative frequencies of all non‐core reactions (i.e. the reactions that are absent in at least one stage‐specific model). The numbers in the two right‐most columns represent the core set of reactions (shared by all stage‐specific models) and the total set of reactions for the respective pathway (core + non‐core).

**Table S1** Properties of all reactions in the *Phytophthora infestans* model.

**Table S2** Gap‐filling solutions, drain reactions, candidate metabolites and essential reactions in the *Phytophthora infestans* model.

**File S1** The *Phytophthora infestans* model in SBML format.
