# Supplementary figures and images for: Genome‐wide characterization of Phytophthora infestans metabolism: a systems biology approach
Source: Mol Plant Pathol. 2018 Jan 30;19(6):1403–13. doi: 10.1111/mpp.12623 (PMC6638193; doi:10.1111/mpp.12623)

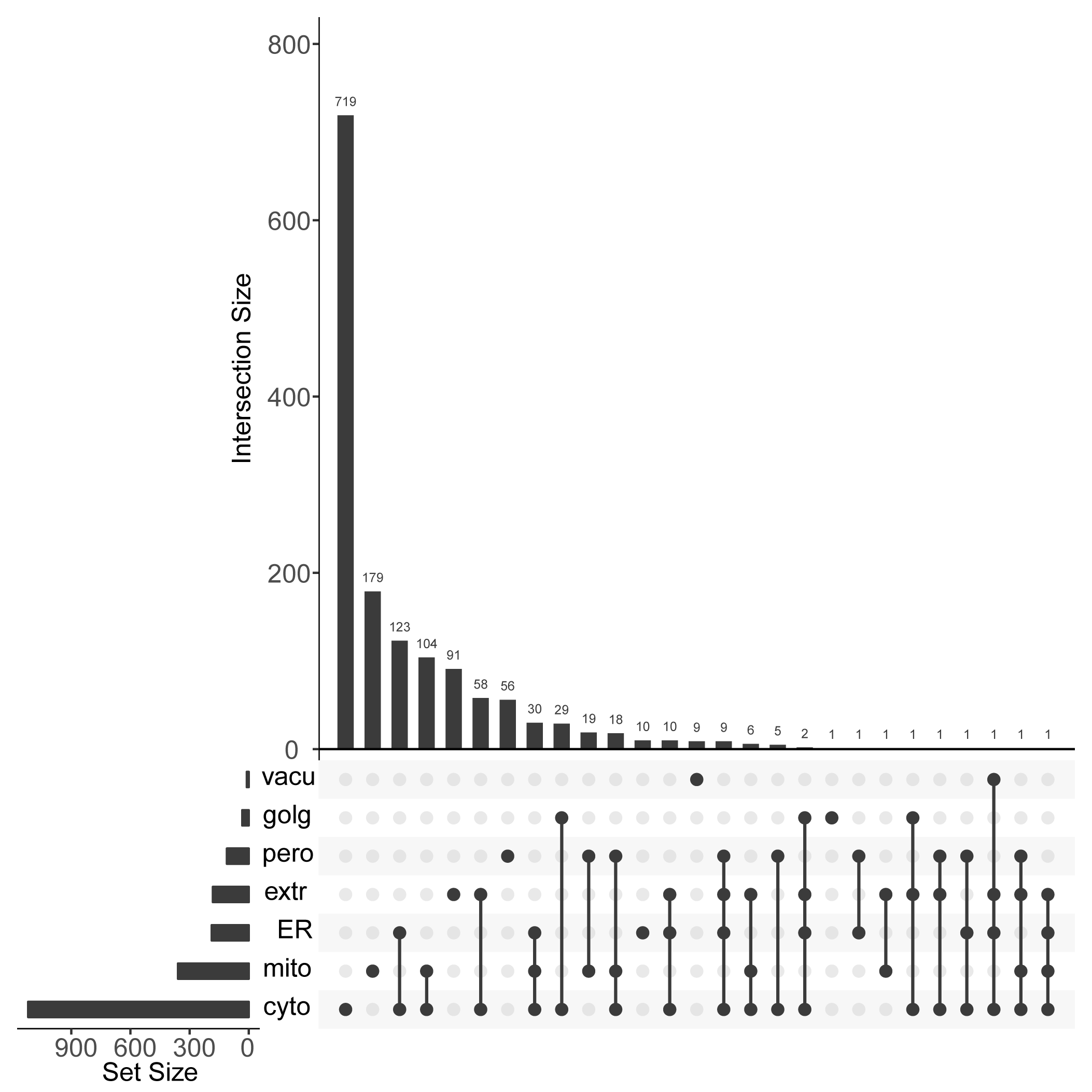

Supplement: Supplementary file 1 — Fig. S1 Reactions in the Phytophthora infestans model per subcellular compartment, and overlap in reaction content between different subcellular compartments. These include the cytosol (cyto), mitochondrion (mito), extracellular space (extr), endoplasmic reticulum (ER), peroxisome (pero), Golgi complex (golg) and vacuole (vacu). The bars at the bottom left show the total numbers of reactions in each subcellular compartment. The connected bullets indicate the compartments that are compared, and the bars in the graph represent the number of reactions (intersection size, y‐axis) that overlap between the compartments. [file MPP-19-1403-s001.tif]

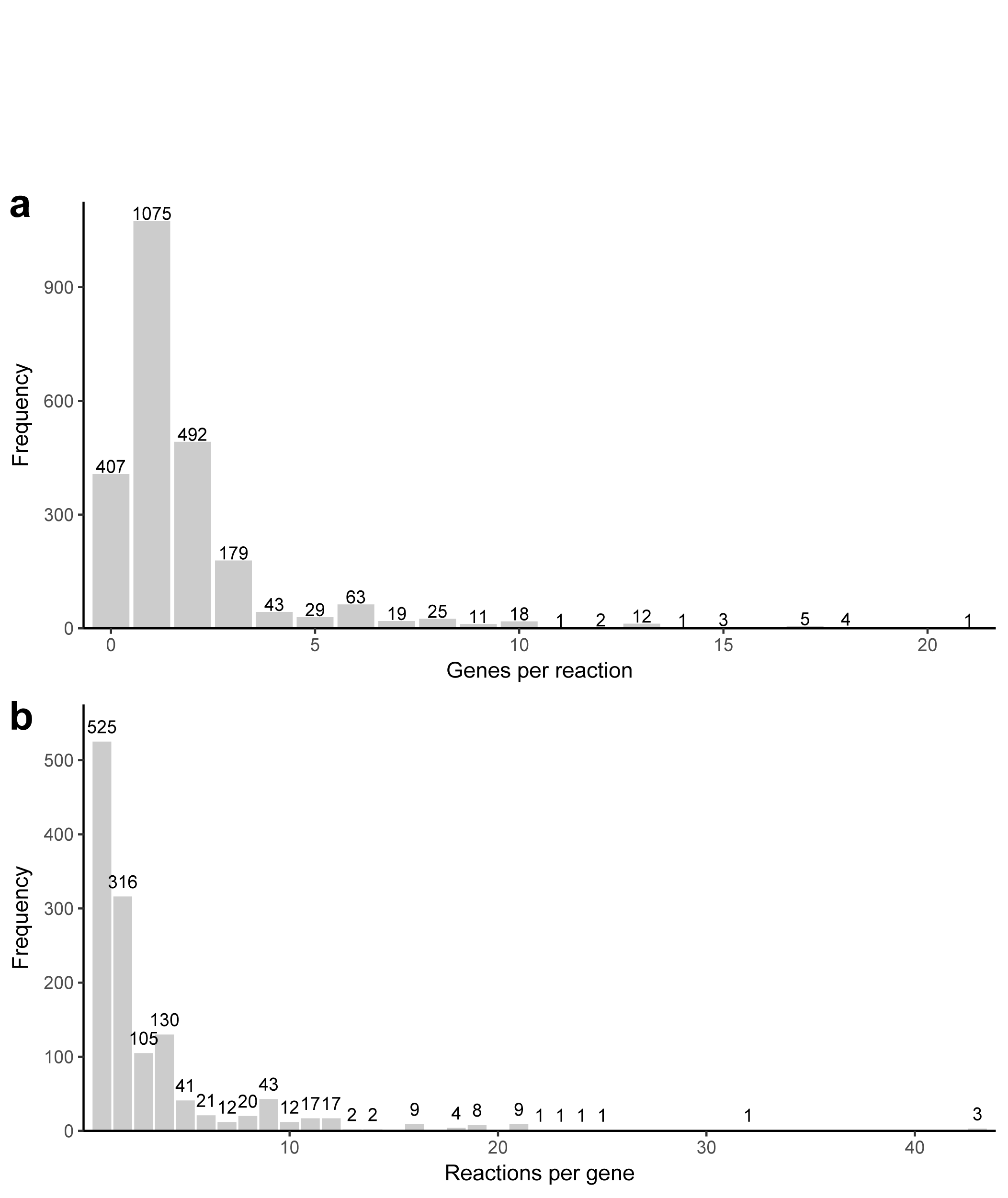

Supplement: Supplementary file 2 — Fig. S2 Frequencies of gene numbers per reaction (a) and reaction numbers per gene (b) in the Phytophthora infestans model. [file MPP-19-1403-s002.tif]

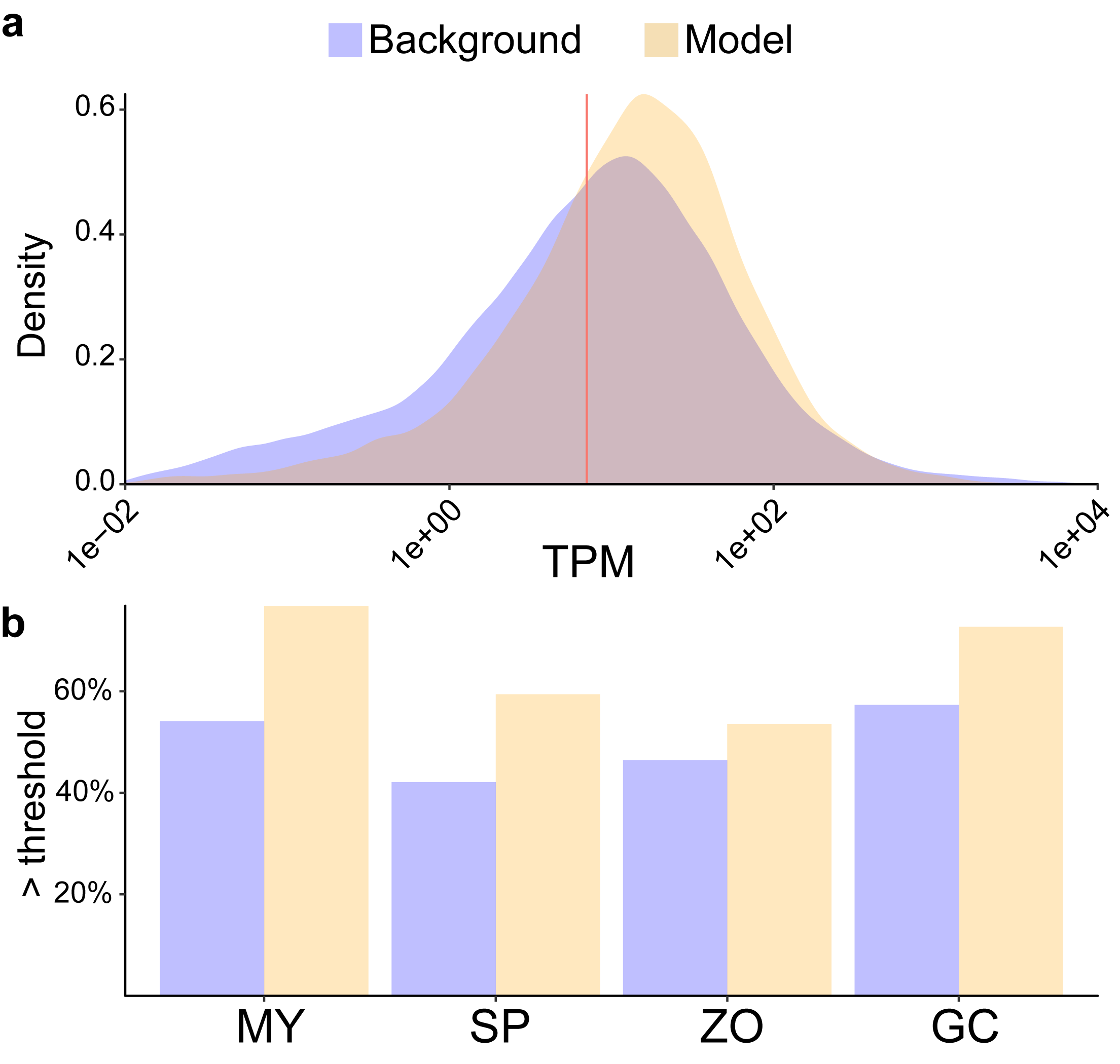

Supplement: Supplementary file 3 — Fig. S3 Transcriptome data of Phytophthora infestans in relation to stage‐specific metabolic models. (a) Distributions of transcripts per million (TPM) expression values of all P. infestans genes (background) and the genes in the model, combined from four life stages. The red line indicates the TPM threshold set to distinguish expressed/non‐expressed genes in the model. (b) The percentages of all P. infestans genes (background) and the genes in the model for which gene expression in each life stage exceeds the TPM threshold. MY, mycelium; SP, sporangia; ZO, zoospores; GC, germinating cysts. [file MPP-19-1403-s003.tif]

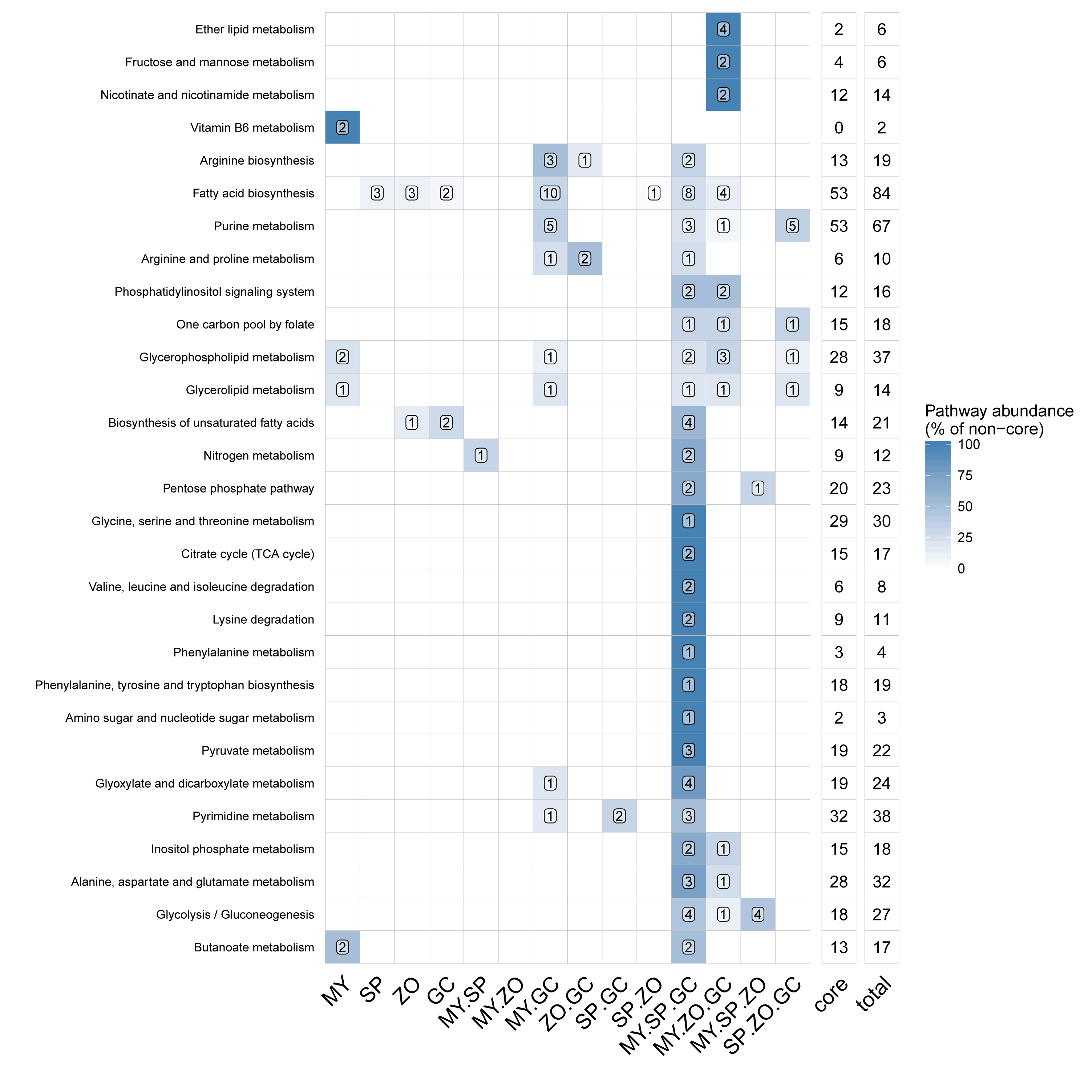

Supplement: Supplementary file 4 — Fig. S4 Numbers of reactions per KEGG pathway that are shared between the Phytophthora infestans life stage‐specific models of mycelium (MY), sporangia (SP), zoospores (ZO) and germinating cysts (GC). The colours of the tiles scale to the relative frequencies of all non‐core reactions (i.e. the reactions that are absent in at least one stage‐specific model). The numbers in the two right‐most columns represent the core set of reactions (shared by all stage‐specific models) and the total set of reactions for the respective pathway (core + non‐core). [file MPP-19-1403-s004.tif]
